# Data from the Multi-Sensitivity Study: Sensory and Rejection Sensitivity, Mental Health, Substance Use, Discrimination, and Social Support

**DOI:** 10.5334/jopd.155

**Published:** 2026-11-02

**Authors:** Charlie Rioux, Megan E. Waldron, Delaney C. Fulp

**Affiliations:** 1 Department of Interdisciplinary Human Sciences, Texas Tech University, United States; 2 Department of Psychology, University of Oklahoma, United States

**Keywords:** Sensory sensitivity, Sensory profile, Differential susceptibility, Mental health, Social environment

## Abstract

Data from the Multi-Sensitivity Study includes sociodemographic factors, sensory processing sensitivity (a.k.a, highly sensitive personality), the Adult Sensory Profile, rejection sensitivity, mental health (distress, well-being, alcohol/cannabis use), discrimination, and social support (informational, emotional, and companionship). The data (*n* = 617, United States) was collected through an online cross-sectional self-report survey on Prolific. The Prolific representative sample option was used, stratifying based on sex, age, and ethnicity. The primary and processed data are available on OSF and have reuse potential for clinical and social psychology research, psychometrics research, methodological research on crowdsourcing for participant recruitment, and for use in statistics courses.

## (1) Background

The environmental sensitivity framework (Pluess, 2015) integrates several models aiming to explain differences in individuals’ sensitivity to positive and/or negative environmental influences. One of those models, *sensory processing sensitivity*, suggests that some individuals have a highly sensitive personality (Aron et al., 2012; Aron & Aron, 1997). This temperament trait is thought to stem from the central nervous system and captures sensitivity to positive and negative environments, where being high on sensory processing sensitivity involves a higher awareness of sensory stimulation, behavioral inhibition, cognitive processing of social, emotional, and physical cues, as well as emotional and physiological reactivity (Aron et al., 2012; Aron & Aron, 1997).

Sensory processing sensitivity has been found to be associated with many factors, including mental health, other personality and temperament traits (e.g., Five-Factor model (McCrae & John, 1992), Rothbart’s temperament model (Rothbart & Derryberry, 1981), Eysenck’s personality model (Eysenck, 1967), Gray’s reinforcement sensitivity model (Gray, 1982)), genotype, and neural/neurocognitive mechanisms (Falkenstein et al., 2025; Greven et al., 2019; Lionetti et al., 2019). The associations of sensory processing sensitivity with some other types of individual sensitivity factors (e.g., rejection sensitivity, sensory profile), however, have not been examined.

Another model of environmental sensitivity (Pluess, 2015), differential susceptibility, can be examined in conjunction with sensory processing sensitivity. The differential susceptibility model also suggests that individuals differ in their sensitivity to their environment, but with a focus on person-environment interactions. Specifically, sensitive individuals would have poorer psychological outcomes than non-sensitive individuals when exposed to negative environments and better outcomes than non-sensitive individuals when exposed to positive environments (Belsky & Pluess, 2009, 2013). Differences in sensitivity to negative environments only is captured by diathesis-stress, while differences in sensitivity to positive environments only is captured by vantage sensitivity (Monroe & Simons, 1991; Pluess, 2015; Pluess & Belsky, 2013). Because it is thought to capture sensitivity to positive and negative environmental stimuli, sensory processing sensitivity may be a differential susceptibility factor within person-environment interactions, but few studies have examined these interactions. For instance, a recent scoping review found that only two studies examined sensory processing sensitivity when testing differential susceptibility, diathesis-stress, and vantage sensitivity in the prediction of adult mental health (Nhem et al., 2026).

Accordingly, the collection of data presented in this paper had two main aims: (1) examine associations of sensory processing sensitivity with the sensory profile and rejection sensitivity to better understand how sensory processing sensitivity manifests; and (2) examine interactions between sensory processing sensitivity and the social environment in the prediction of adult mental health. While these were the data collection’s main objectives, this resulted in the collection of a variety of sociodemographic, individual characteristic, social, and mental health factors, which can be of potential use for future research and secondary data analyses.

## (2) Methods

### 2.1 Study design

Data was collected for this project using a cross-sectional online survey. Participants were recruited through Prolific and provided with a link to access an online survey hosted on Research Electronic Data Capture (REDCap (Harris et al., 2009, 2019)) at the University of Oklahoma Health Sciences Center. Prior to beginning the study, all individuals were shown a consent form that provided an overview of the study. If individuals chose not to consent, the survey would shut down. To be eligible to participate, participants needed to: (1) be at least 18 years old; (2) currently reside in the United States; and (3) be able to read English. Eligibility was determined by Prolific based on user data and only eligible participants were offered participation in the study. Eligible participants answered sociodemographic questions, followed by questionnaires on rejection sensitivity, the Sensory Profile, discrimination, sensory processing sensitivity, alcohol use, cannabis use, well-being, psychological distress, and social support.

### 2.2 Time of data collection

Data was collected between November 6 and November 9, 2023.

### 2.3 Location of data collection

Data was collected in the United States. The geographical distribution of the sample can be found in Figure 1; participants were from every state, except for Wyoming and North Dakota.

**Figure 1:**
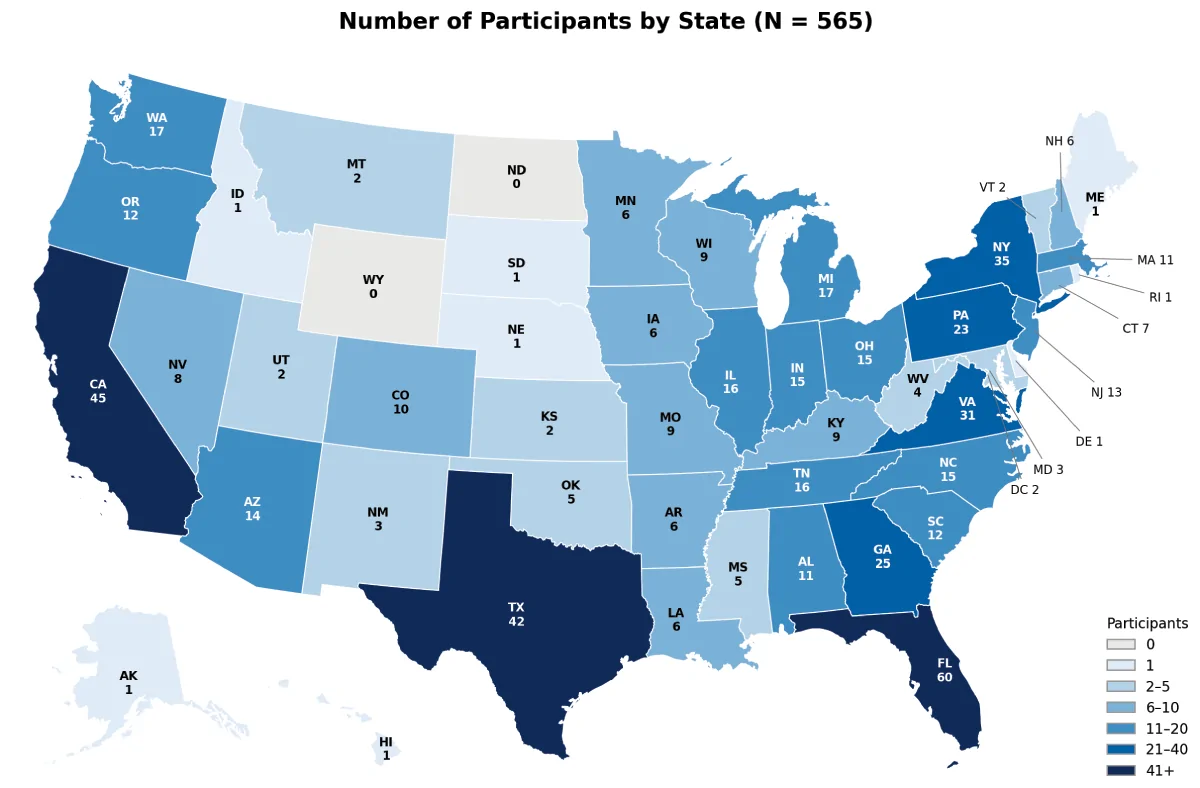
Geographical distribution of study participants.

### 2.4 Sampling, sample and data collection

Participants were recruited through Prolific, which has been found to yield high data quality compared to other crowdsourcing platforms, with participants showing higher attentiveness (Albert & Smilek, 2023; Douglas et al., 2023; Peer et al., 2021). Participant eligibility was determined by the Prolific pre-screening tool based on user data and included being at least 18 years old, currently residing in the United States, and being able to read English. The Prolific standard representative United States sample option was used, which stratifies the sample based on Census sex, age, and ethnicity data. Stratified representative sampling as implemented by Prolific is done in 50 subgroups with two strata for sex (female, male), five strata for age (18–27, 28–37, 38–47, 48–57, and 58+), and five strata for race (White, Mixed, Asian, Black, and Other). Prolific allows participation through a first-come first-serve procedure, where a participant was advertised the study if a space remained in their subgroup. In the present work, a sample size of 600 was targeted. To ensure the study was working properly on the platform, it was first run with 10 participants without the representative sample feature (which requires at least 300 participants). The study was then run with a target of 590 participants using the representative sample feature. Once the survey was completed, participants were redirected to Prolific, which registered the task as complete on the platform. Participants received a compensation of $5USD upon completion.

The survey was completed by 617 participants, with 3 participants completing only part of the survey and 14 completing the full survey, but in more time than allowed by Prolific. Specifically, Prolific stops recruitment once the target number of participants (600) is reached, with this number only including participants who complete the full survey and enter their completion code in Prolific in the allotted time. Prolific automatically sets a maximum allowed time for all studies based on the estimated completion time. The maximum allowed time is designed to give plenty of time to participants while giving spots to new participants if someone is inactive for a long time. In this study, 14 participants timed out on Prolific after 67 minutes. The expected completion time was set at 20 minutes and the final median completion time was 18 minutes 35 seconds.

All 617 participants provided consent and were thus included for potential inclusion in the final dataset. From this sample, unreliable data (from inattention and careless responding) was identified through two attention checks, negative answers to a question asking participants if they were at least 18 years old (indicating a mismatch with Prolific user data used for eligibility), and completion times under nine minutes, which were considered impossible based on survey testing. This resulted in the removal of 52 participants, resulting in a final sample of 565 participants. The sample demographics can be found in Table 1.

**Table 1:** Sample demographics.


VARIABLE	n (%)

Age (range = 18–94; Mean (SD))	46.65 (15.84)

Sex assigned at birth	

Female	286 (50.6%)

Male	273 (48.3%)

Intersex	1 (0.2%)

Missing	5 (0.9%)

Gender	

Woman	276 (48.8%)

Man	271 (48.0%)

Non-binary, agender, or gender queer	17 (3.0%)

Missing	1 (0.2%)

Ethnicity	

Black	76 (13.5%)

Latino/a/x	21 (3.7%)

Asian or Asian American	34 (6.0%)

American Indian or Native American	3 (0.5%)

White	417 (73.8%)

Multiracial or Multiethnic	11 (1.9%)

Missing	3 (0.5%)

Immigrant status	

First generation immigrant	34 (6.0%)

Second generation immigrant	69 (12.2%)

Participant and their parents born in the US	458 (81.1%)

Missing	4 (0.7%)

Education	

High school diploma or less	184 (32.6%)

Technical/trade/vocational training diploma	33 (5.8%)

Associate degree or undergraduate certificate	68 (12.0%)

Bachelor’s degree	181 (32.0%)

Masters degree	71 (12.6%)

PhD	13 (2.3%)

Professional degree (e.g., MD, JD, DSS…)	11 (1.9%)

Missing	4 (0.7%)

Marital status	

Married	192 (34.0%)

In a common-law/domestic partnership	16 (2.8%)

Living with a partner	64 (11.3%)

In a relationship but living separately	34 (6.0%)

Single, never married	165 (29.2%)

Single, widowed	12 (2.1%)

Single, divorced	69 (12.2%)

Single, separated	9 (1.6%)

Missing	4 (0.7%)

2022 pretax household income (USD)	

Less than $20,000	67 (11.9%)

$20,000–39,999	115 (20.4%)

$40,000–69,999	140 (24.8%)

$70,000–99,999	107 (18.9%)

$100,000–124,999	58 (10.3%)

$125,000–149,999	30 (5.3%)

$150,000–174,999	11 (1.9%)

$175,000–199,999	11 (1.9%)

$200,000 or more	24 (4.2%)

Missing	2 (0.4%)

Type of community lived in	

Large city	118 (20.9%)

Suburb near a large city	230 (40.7%)

Town or small city	139 (24.6%)

Rural area	75 (13.3%)

Missing	3 (0.5%)


*Note*. Percentages rounded to one decimal place; totals may differ slightly from 100% due to rounding.

### 2.5 Materials/Survey instruments

Questionnaires below are listed in the order of the online survey as seen by participants. A copy of the study questionnaires is available with the data, see Section 3.

#### 2.5.1 Sociodemographic questionnaire

The survey began with questions on demographic factors. The following variables were assessed: state currently lived in, age, country of birth, country of both parents’ birth, race/ethnicity, highest level of education attained, relationship status, partner’s gender identity, 2022 pre-tax household income, daily activities and responsibilities, present religion, type of community lived in (i.e., large city, suburb near a large city, town or small city, or rural area), gender identity, sex assigned at birth, and sexual orientation.

#### 2.5.2 Rejection sensitivity

Rejection sensitivity was measured using the Adult Rejection Sensitivity Questionnaire (Berenson et al., 2009). This questionnaire consists of nine hypothetical interpersonal interactions where rejection by a significant other is possible (e.g., “After a bitter argument, you call or approach your significant other because you want to make up”). Each situation is followed by two items. The first item measures the level of concern/anxiety participants felt about the outcome of each situation (e.g., “How concerned or anxious would you be over whether or not your significant other would want to make up with you?”), rated on a Likert scale going from 1 (very unconcerned) to 6 (very concerned). The second item measures the level of acceptance expectancy, i.e., the perceived likelihood that the significant other would *not* respond with rejection (e.g., “I would expect that they would be at least as eager to make up as I would be”), rated on a Likert scale going from 1 (very unlikely) to 6 (very likely). The total score of rejection sensitivity was computed. To do so, rejection sensitivity for each situation was first calculated by multiplying the level of rejection concern by the reverse of the level of acceptance expectancy. Second, the mean of the nine situation scores was computed where higher scores reflect higher rejection sensitivity. Reliability was good in this sample (α = 0.79).

#### 2.5.3 Sensory profile

Participants completed the Adolescent/Adult Sensory Profile^™^ (Brown et al., 2001; Brown & Dunn, 2002). The Sensory Profile measures the four quadrants of Dunn’s Model of Sensory Processing (Dunn, 1997, 2001; see Figure 2): low registration, sensation seeking, sensory sensitivity, and sensation avoiding, which are based on the intersection of two continua, neurological threshold and self-regulation. Neurological threshold corresponds to how much stimulus is needed for someone to notice and respond and ranges from low (sensitization) to high (habituation). Self-regulation is a behavioral continuum corresponding to how passively or actively individuals react to stimuli and ranges from passive (reactions are in accordance with the neurological threshold) to active (reactions aim to counteract neurological thresholds). The combination of the neurological and behavioral continua delineates the four quadrants of sensory processing (see Figure 1). The first quadrant, *low registration*, captures responding to a high threshold with passive self-regulation, where stimuli are passively disregarded (missed or responded to with a delay). The second quadrant, *sensation seeking*, captures responding to a high neurological threshold with active self-regulation, additional stimuli are actively sought because they are pleasurable. The third quadrant, *sensory sensitivity*, captures responding to a low neurological threshold with passive self-regulation, where stimuli are difficult to ignore and responded to with passive discomfort. The fourth and last quadrant, *sensory avoiding*, captures responding to a low neurological threshold with active self-regulation, exposure to stimuli is actively limited because they are overwhelming (Brown & Dunn, 2002). The Sensory Profile aims to measure the sensory history of individuals in all four quadrants independently. As such, each quadrant is assessed on its corresponding continua, and individuals can be high, average, or low in all four patterns.

**Figure 2:**
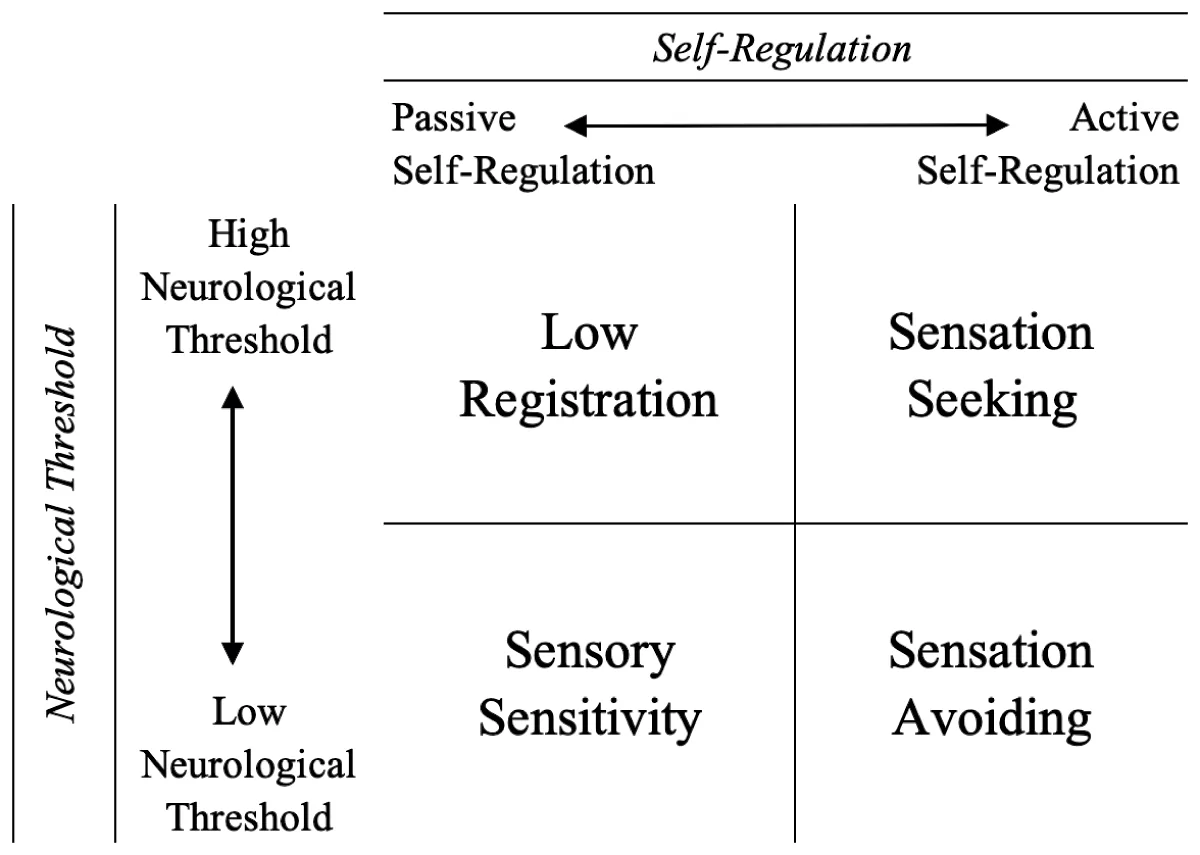
Dunn’s model of sensory processing consisting of two continua (neurological threshold and self-regulation) delineating four quadrants. From Rioux et al (2025), published under Creative Commons Attribution 4.0 International License.

The Adolescent/Adult Sensory Profile^™^ consists of 60 items describing everyday sensory experiences, with 15 items per quadrant, rated from 1 (almost never) to 5 (almost always). Within each quadrant, items cover six domains: taste/smell processing, movement processing, visual processing, touch processing, activity level, and auditory processing. The instrument was scored based on the user manual (Brown & Dunn, 2002). Raw continuous scores were computed for each quadrant using the sum of the corresponding items (range 15–75). In this sample, reliability was low for sensation seeking but good for the other three quadrants (low registration α = 0.84; sensation seeking α = 0.69; sensory sensitivity α = 0.82; sensation avoiding α = 0.81).

Categorical scores were also computed for each quadrant based on the established clinical cut-offs (Brown & Dunn, 2002), which differ based on the quadrant and participant age (see Table 2). There are five groups per quadrant: Much less than most people; Less than most people; Similar to most people; More than most people; and Much more than most people.

**Table 2:** Cut-off scores for the sensory profile quadrants.


QUADRANT	CLASSIFICATION	CUT-OFF SCORES	

		18–64 YEARS OLD	65+ YEARS OLD

Low registration	Much less than most people	15–18	15–19

	Less than most people	19–23	20–26

	Similar than most people	24–35	27–40

	More than most people	36–44	41–51

	Much more than most people	45–75	52–75

Sensation seeking	Much less than most people	15–35	15–28

	Less than most people	36–42	29–39

	Similar than most people	43–56	40–52

	More than most people	57–62	53–63

	Much more than most people	64–75	64–75

Sensory sensitivity	Much less than most people	15–18	15–18

	Less than most people	19–25	19–25

	Similar than most people	26–41	26–41

	More than most people	42–48	42–48

	Much more than most people	49–75	49–75

Sensation avoiding	Much less than most people	15–19	15–18

	Less than most people	20–26	19–25

	Similar than most people	27–41	26–42

	More than most people	42–49	43–49

	Much more than most people	50–75	50–75


#### 2.5.4 Discrimination

Discrimination was measured using the short version of the Everyday Discrimination Scale (Williams et al., 1997). This questionnaire includes five items where participants indicate the frequency in which certain discriminatory experiences happen to them in their day-to-day lives on a 6-point scale (5 = almost every day, 4 = a few times a week, 3 = a few times a month, 2 = a few times a year, 1 = less than once per year, 0 = never). Items capture how the participant is treated (e.g., “you receive poorer service than others at restaurants or stores”), how they believe others perceive them (e.g., “people act as if they are afraid of you”), and whether they had been threatened or harassed. Total discrimination scores were computed by summing all items. Reliability was good in this sample (α = .86). Participants who indicated that they had experienced at least one of the items “a few times a year” or more were also prompted to answer a follow-up question in which they were asked to report what they perceived as the primary reason for their discrimination.

#### 2.5.5 Sensory processing sensitivity

Sensory processing sensitivity was measured using the Highly Sensitive Person Scale-12 items (Pluess et al., 2023). The items are rated on a Likert scale from 1 (not at all) to 7 (extremely). Mean scores of all items were computed for the total scale score. Reliability was good in this sample (Cronbach’s α = .81). A categorical score was also computed (low sensitivity, average sensitivity, high sensitivity) where the bottom 30%, middle 60%, and top 30% were used as suggested during scale development (Pluess et al., 2023). Subscale mean scores were computed for low sensory threshold (3 items), ease of excitation (5 items), and aesthetic sensitivity (4 items). Low sensory threshold captures how easily one is aroused by subtle stimuli; ease of excitation captures how easily one reaches a state of overwhelm via exposure to stimuli; and aesthetic sensitivity captures levels of appreciation and pleasure experienced from aesthetics (e.g., art, beauty).

#### 2.5.6 Alcohol use

For alcohol use questions, participants were first provided with instructions defining a drink. Specifically, the instructions stated “The next questions are about drinks of alcoholic beverages. By a “drink,” we mean a can or bottle of beer, a glass of wine or a wine cooler, a shot of liquor, or a mixed drink with liquor in it. We are not asking about times when you only had a sip or two from a drink. Definition of a standard drink: One 12oz bottle of beer, one 5oz glass of wine, 1.5oz of liquor as a shot or in a mixed drink.” A figure was provided, showing what a standard drink of different types of alcohol looks like.

Participants were first asked if they had at least 1 drink of alcohol in their life, not counting small tastes or sips. If they answered no, they skipped the rest of the alcohol questionnaire. The second question assessed *age of onset* by asking how old they were the first time they had a drink of an alcoholic beverage, not including sips. Answer options ranged from 12 years of younger to 21 years or older, with every possible year in between.

This was followed by the Alcohol Use Disorder Identification Test – Consumption (AUDIT-C (Bush, 1998)), consisting of three questions about alcohol use within the past year. The first question assesses *alcohol use frequency* (Never, Monthly of less, 2–4 times a month, 2–3 times a week, 4 or more times a week; participants answering “Never” skipped the next two questions). The second question assesses alcohol quantity (1–2, 3–4, 5–6, 7–9, 10 or more). The last question assesses *binge drinking frequency*, asking how often participants had six or more drinking on one occasion (Never, Less than monthly, Monthly, Weekly, Daily or Almost daily). The AUDIT-C score was computed using a sum of all items, with participants who never had alcohol having a score of 0. Reliability of the AUDIT-C was acceptable in this sample (α = .72).

In addition to the raw scores and the AUDIT-C score, alcohol use frequency, binge drinking frequency, and alcohol quantity-by-frequency were computed. Alcohol use and binge drinking frequencies are based on the first question of the AUDIT, with participants who did not drink alcohol having a score of 0. Alcohol quantity-by-frequency consists in the product of the first two AUDIT questions, with participants who did not drink alcohol having a score of 0.

#### 2.5.7 Cannabis use

For cannabis use questions, participants were first provided with instructions defining cannabis products. Specifically, instructions stated “The next questions ask about cannabis (marijuana) products. This includes dried plant materials (bud/flower), cannabis concentrates (wax, shatter, hash oil, dabs, etc.), and cannabis-infused edibles (including beverages). It does not include products containing only cannabidiol (CBD).”

Participants were first asked if they had ever used cannabis products. If they answered no, they skipped the rest of the cannabis questionnaire. The second question assessed *age of onset* by asking how old they were the first time they had cannabis products. Answer options ranged from 12 years of younger to 21 years or older, with every possible year in between.

This was followed by the first two questions of the Cannabis Use Disorder Identification Test Short-Revised (CUDIT-R; (Adamson et al., 2010)). The first question assesses the frequency of use within the last six months (from 0 = Never to 4 = Four times or more a week). A cannabis use frequency variable was computed based on this question, with participants who never used cannabis having a score of 0. The second question assessed hours stoned on typical days of use (from 0 = Less than one to 4 = Seven or more). Cannabis quantity-by-frequency was computed using the product of the first the two CUDIT questions, with participants who never used cannabis having a score of 0.

#### 2.5.8 Psychological well-being

Well-being was measured using the Mental Health Continuum short-form (Lamers et al., 2011), a 14-item measure of general well-being that captures the frequency of experiences of positive mental health across three dimensions: emotional well-being, social well-being, and psychological well-being. Items are answered on a 6-point Likert scale ranging from 0 (“Never”) to 5 (“Every day”). A sum score of all items was computed, ranging from 0 to 70 with higher scores indicating greater psychological well-being. Reliability was high in this sample (α = .94).

#### 2.5.9 Psychological distress

Distress was measured using the Kessler Psychological Distress Scale (Kessler et al., 2002), a 10-item self-report questionnaire that measures overall psychological distress, including anxiety and depression symptoms. The scale assessed the frequency in which symptoms were experienced in the last four weeks on a 5-point Likert scale ranging from 1 (“None of the time”) to 5 (“All of the time”). A sum score of all items was computed, ranging from 10 to 50 with higher scores indicating greater psychological distress. Reliability was high in this sample (α = .93). A categorical variable was also computed using the clinical cutoffs for this scale (Victorian Government Department of Human Services, 2022), where a score of 10–19 indicates a likelihood to not have a disorder, 20–24 is likely to have a mild disorder, 25–29 is likely to have a moderate disorder, and 30–50 is likely to have a severe disorder.

#### 2.5.10 Social support

Social support was measured using the three quality of social support scales from the Patient-Reported Outcomes Measurement Information System (PROMIS® (Hahn et al., 2010)). The PROMIS® Informational Support – Short Form 4a assesses the availability of others to obtain beneficial information, advice, or suggestions when needed, with four items rated from 1 (Never) to 5 (Always). A sum score of all items was computed, ranging from 4 to 20 with higher scores indicating higher levels of informational support. Reliability was high in this sample (α = .94).

The PROMIS® Emotional Support – Short Form 4a assesses the availability of individuals to talk to and confide in, with four items rated from 1 (Never) to 5 (Always). A sum score of all items was computed, ranging from 4 to 20 with higher scores indicating higher levels of emotional support. Reliability was high in this sample (α = .96).

The PROMIS® Companionship Support – Short Form 4a assesses the availability of individuals to do activities, have fun, and/or relax with, with four items rated from 1 (Never) to 5 (Always). A sum score of all items was computed, ranging from 4 to 20 with higher scores indicating higher levels of emotional support. Reliability was high in this sample (α = .96).

### 2.6 Quality Control

Best-practice recommendations for online crowdsourcing (Aguinis et al., 2021; Ng et al., 2025) were employed, including a reCAPTCHA verification and attention checks. Two attention checks were included using instructed response items (Gummer et al., 2021). Specifically, “Because it is important for the quality of our survey data, we are checking if you are paying attention. Please select Occasionally on this question” was included between items 2 and 3 of the Adult Sensory Profile (section 2.5.3) and “Paying attention to the survey is important for the quality of the data, so we need to check your attention. Please select 6 on this question” was included as the last question of the highly sensitive person scale (section 2.5.5). Unreliable data (from inattention, careless responding, and web robots) was identified through these attention checks as well as negative answers to a question asking participants if they were at least 18 years old (indicating a mismatch with Prolific user data used for eligibility), and completion times under nine minutes, which were considered impossible based on survey testing (see section 2.4). The reliability of questionnaires/scales within the study sample is provided using Cronbach’s alphas (see section 2.5).

During scale computations, scales available in the processed data were checked for univariate outliers by examining the combination of standardized values (Z-scores) exceeding ± 3.29 and being out of the distribution of other scores on the variable (Tabachnick & Fidell, 2019). Identified univariate outliers’ raw score were windsorized to be a unit larger or smaller than the next most extreme score (Tabachnick & Fidell, 2019). This process is included in the provided syntax files (see section 3). Two processed datafiles are provided for reuse: One with computed, non-windsorized scale scores, and one with these windsorized scores.

### 2.7 Data anonymisation and ethical issues

Ethics approval was granted by the University of Oklahoma Norman Campus Institutional Review Board (IRB#16621). All participants provided electronic informed consent prior to answering study questionnaires by reviewing and agreeing to the project’s consent form on REDCap. Consent included de-identified data being made be made available on online data repository platforms. The consent form is provided in the study materials of the OSF project (see section 3). All participants were given a random ID for the dataset to protect their identity.

A few variables had to be removed from the public dataset to anonymise the data due to their potential for participant identification through cross tabulation. This includes location data and variables with low counts (under *n* = 10).

Specifically, *state of residence* was removed due to a large proportion of states having less than 10 participants. The original *age* variable, a continuous variable with final values going from 18 to 94, was removed due to many ages having low participant counts. A categorical age variable is included in the processed dataset, with 21 and under, followed by decade-based groups (22–29, 30–39…) up to 70+). The number of participants under 21 and over 80 was too small for them to be in their own group. *Country of birth* of both participants and their parents were removed due to low counts. An immigration status variable was computed and is included in the processed dataset, capturing groups for first generation immigrant, second generation immigrant, and participants + both parents born in the USA. Regarding *race/ethnicity*, the questionnaire included an open-ended question asking participants the race/ethnicity they identify with, followed by a categorical answer (Table 1). The open-ended question was removed due to low counts (since most answers are unique). The categorical answer was removed from the raw data because the category American Indian had too few participants. In the processed dataset, race/ethnicity is provided, where American Indian was changed to be missing and the other categories were kept intact. There were participants with missing data on this variable, thus American Indian participants are not identifiable through missingness. *Relationship status* was removed due to the number of separated participants being under 10. In the processed data, the variable was recoded so that separated and divorced participants are in the same category. *Partner’s gender identity* was removed due to the number of gender-diverse participants being too low. In the processed data, gender-diverse options were changed to be missing, with Man/Woman remaining. There were participants with missing data on this variable, thus gender-diverse partners are not identifiable through missingness. *Participant’s gender identity* was removed due to some subcategories of gender-diverse participants being too low. In the processed data, the variable was recoded to have one gender-diverse category, which includes participants who reported identifying as Non-binary, Agender, or Gender Queer. *Sex assigned at birth* was removed due to the number of intersex participants being too low. In the processed data, the intersex option was changed to be missing. There were participants with missing data on this variable, thus intersex participants are not identifiable through missingness. Finally, *sexual orientation* was removed due to some identities having low counts. In the processed data, identities were combined. Specifically, heteroflexible was combined with the bisexual/pansexual/polysexual category and demisexual was combined with the asexual/nonsexual category.

### 2.8 Existing use of data

As of Fall 2026, the data has been used for three projects:

**1.** Rioux, C., Waldron, M. E., Fulp, D. C., Personette, C. M. & Nhem, M. K. (2026). Sensory profile of the highly sensitive personality. *Personality and Individual Differences*. *255*. Article 113710. https://doi.org/10.1016/j.paid.2026.113710

This project examined associations between sensory processing sensitivity (section 2.5.5) and the sensory profile (section 2.5.3).

**2.** Nhem, M., Personette, C., Tasasiz, B. & Rioux, C. Environmental sensitivity to discrimination in the prediction of adult mental health. Undergoing peer review. The preprint can be found at https://doi.org/10.31219/osf.io/xw6pv_v1

This project examined the moderation of the association between everyday discrimination (section 2.5.4) and mental health (sections 2.5.8 and 2.5.9) by sensory processing sensitivity (section 2.5.5).

**3.** Stephens, J. I. Hinkle, L. & Rioux, C. Relationship between sensory processing sensitivity and rejection sensitivity and their association with mental health. Undergoing peer review. The preprint can be found at https://doi.org/10.31234/osf.io/wdqrg_v1

This project examined associations between sensory processing sensitivity (section 2.5.5) and rejection sensitivity (section 2.5.2) and whether both types of sensitivity uniquely predict mental health (sections 2.5.8 and 2.5.9).

## (3) Dataset description and access

### 3.1 Repository location

https://doi.org/10.17605/OSF.IO/AVKP5.

### 3.2 Object/file name

See Table 3.

**Table 3:** OSF project file descriptions for primary and processed data.


FOLDER NAMES	FILE NAMES	DESCRIPTION	DATA TYPE

Raw data	SensitivityStudy_OpenRawData.sav SensitivityStudy_OpenRawData.csv	Primary data file, excluding identifying information (section 2.7)	Primary data

Processed data	SensitivityStudy_ProcessedData_NOT-Windsorized.sav SensitivityStudy_ProcessedData_NOT-Windsorized.csv SensitivityStudy_ProcessedData_Windsorized.sav SensitivityStudy_ProcessedData_Windsorized.csv	Data following sample processing (section 2.4), scale computations (section 2.5 and 2.7), and Quality control (section 2.6)	Processed data

	SensitivityStudy_Syntax.sps SensitivityStudy_Syntax.txt	Syntax to make processed data from primary data.	Code

	SensitivityStudy_Output.spv SensitivityStudy_Output.htm	Output from SensitivityStudy_Syntax.sps	Output

Study materials	SensitivityStudy_ConsentForm.pdf	Consent form completed by participants.	Materials

	SensitivityStudy_ProlificStudyDetails.pdf	Advertisement posted on Prolific to recruit participants.	Materials

	SensitivityStudy_DataDictionary.ods	Data dictionary	Codebook

	SensitivityStudy_REDCapDataDictionary.csv	Data dictionary from REDCap	Materials

	SensitivityStudy_Questionnaires.pdf	PDF of study questionnaire completed on REDCap	Materials


### 3.3 Data type

Table 3 provides a summary of the data files. Each folder contains README text files that also summarize the files within the folder, labels of key variables, and data restrictions.

### 3.4 Format names and versions

All data are available in SPSS and comma separated values (CSV) formats.

### 3.5 Language

American English

### 3.6 License

CC-By Attribution 4.0 International

### 3.7 Limits to sharing

There is no limit to sharing.

### 3.8 Publication date

02/09/2025

### 3.9 FAIR data/Codebook

Descriptive metadata was included in the OSF repository, including project description, subjects, and tags. A digital object identifier (DOI) was assigned to the project to support permanent identification. The use of a reputable and widely used repository (OSF) supports accessibility. Downloading the data on OSF does not require identification or authorization procedures, in turn supporting accessibility. The OSF project includes README files to aid in navigating the project folders. SPSS data files were saved and uploaded in CSV format for accessibility. SPSS data files (Table 3) include variable information in the variable labels tab. The variable information was also saved in the data dictionnary as an OpenDocument Spreadsheet (ODS file) (Table 3) for use with the CSV files. The annotated SPSS syntax is also provided in text format and the SPSS output in HTML format to support accessibility of all materials without proprietary software. The study materials, including the consent form, Prolific posting (i.e., study advertisement), questionnaires, and REDCap data dictionary are also provided in the OSF project (Table 3).

## (4) Reuse potential

### 4.1 Key strengths and limitations of the dataset

An important strength of the study is its representativeness in terms of race/ethnicity, age, and sex, which were stratified based on the US Census data. However, due to the overall sample size, some racial-ethnic groups have low counts, which limits analyses examining race/ethnicity specifically. Furthermore, the education level of the sample was higher than the US population. While data was collected using validated scales, a limitation is that all data was self-reported.

### 4.2 Clinical and social psychology research

The dataset has reuse potential for secondary analyses beyond its existing use (section 2.8). This data can be used for clinical psychology research on mental distress, mental well-being, alcohol use, or cannabis use. A range of correlates of mental health can be examined through the present data, including sociodemographic factors, diverse measures of sensory sensitivity, discrimination, and social support. The data is also relevant for social psychology research as a range of individual-level factors (rejection sensitivity, sensory processing sensitivity) and social factors (discrimination, social support) were collected.

The data also potential for examining environmental sensitivity (Pluess, 2015) as captured by sensory processing sensitivity (Aron et al., 2012; Aron & Aron, 1997; Greven et al., 2019). The data can also be used for testing the diathesis-stress, vantage sensitivity, and differential susceptibility theoretical models (Belsky & Pluess, 2009; Monroe & Simons, 1991; Pluess & Belsky, 2013), which suggest different patterns of interaction between individual temperament and environmental factors in the prediction of psychological outcomes.

The data can also be used for individual participant-level meta-analyses (Burke et al., 2017; Sutton et al., 2008), as well as harmonized with other datasets for integrative data analyses (Hussong et al., 2013) related to sensitivity or mental health.

### 4.3 Psychometric research

Several validated scales were collected for this dataset and can be used for psychometrics research evaluating these scales to inform their future use across populations. This may include testing the factorial structure or the scales or factorial invariance (Bontempo & Hofer, 2007; Dimitrov, 2010) based on key sociodemographic variables (e.g., sex, age, education level).

### 4.4 Research on crowdsourcing for participant recruitment

Since the present data was collected via Prolific, it could also be used for methodological research on participant recruitment through crowdsourcing platforms. This includes research examining Prolific participant demographics and psychosocial characteristics or comparing the characteristics of samples recruited through different methods or crowdsourcing platforms. The data could also be used for research testing new data processing and cleaning methods (Chu et al., 2016) for crowdsourced data.

### 4.5 Psychology statistics courses

Student practice with real-world data is recommended to increase student motivation, interest, and engagement in statistics courses (Hall, 2011; Idris, 2018; Smith, 1998; Wiberg, 2009). The present data can be used for advanced undergraduate or graduate statistics courses teaching applied descriptive statistics, t-tests, chi-square tests, correlation, generalized linear modelling, and/or basic psychometrics. The data could also be used for teaching psychometrics, confirmatory factor modelling, and structural equation modelling, since item-level data is available. Since both raw and processed data are provided, the present dataset can be used for classes that begin with data processing/cleaning/wrangling as well as classes that do not cover this part of data analysis.

## References

[1] Adamson, S. J., Kay-Lambkin, F. J., Baker, A. L., Lewin, T. J., Thornton, L., Kelly, B. J., & Sellman, J. D. (2010). An improved brief measure of cannabis misuse: The Cannabis Use Disorders Identification Test-Revised (CUDIT-R). Drug and Alcohol Dependence, 110(1–2), 137–143. 10.1016/j.drugalcdep.2010.02.017 20347232

[2] Aguinis, H., Villamor, I., & Ramani, R. S. (2021). MTurk research: Review and recommendations. Journal of Management, 47(4), 823–837. 10.1177/0149206320969787

[3] Albert, D. A., & Smilek, D. (2023). Comparing attentional disengagement between Prolific and MTurk samples. Scientific Reports, 13(1), 20574. 10.1038/s41598-023-46048-5 PMC10667324 37996446

[4] Aron, E. N., & Aron, A. (1997). Sensory-processing sensitivity and its relation to introversion and emotionality. Journal of Personality and Social Psychology, 73(2), 345–368. 10.1037/0022-3514.73.2.345 9248053

[5] Aron, E. N., Aron, A., & Jagiellowicz, J. (2012). Sensory processing sensitivity: A review in the light of the evolution of biological responsivity. Personality and Social Psychology Review, 16(3), 262–282. 10.1177/1088868311434213 22291044

[6] Belsky, J., & Pluess, M. (2009). Beyond diathesis stress: Differential susceptibility to environmental influences. Psychological Bulletin, 135(6), 885–908. 10.1037/a0017376 19883141

[7] Belsky, J., & Pluess, M. (2013). Beyond risk, resilience, and dysregulation: Phenotypic plasticity and human development. Development and Psychopathology, 25(4pt2), 1243–1261. 10.1017/S095457941300059X 24342838

[8] Berenson, K. R., Gyurak, A., Ayduk, Ö., Downey, G., Garner, M. J., Mogg, K., Bradley, B. P., & Pine, D. S. (2009). Rejection sensitivity and disruption of attention by social threat cues. Journal of Research in Personality, 43(6), 1064–1072. 10.1016/j.jrp.2009.07.007 PMC2771869 20160869

[9] Bontempo, D. E., & Hofer, S. M. (2007). Assessing factorial invariance in cross-sectional and longitudinal studies. In A. D. Ong & M. H. M. Van Dulmen (Eds.), Oxford handbook of methods in positive psychology (pp. 153–175). Oxford University Press. 10.1093/oso/9780195172188.003.0011

[10] Brown, C., & Dunn, W. (2002). Adolescent/adult sensory profile. Psychological Corporation. 10.1037/t56705-000

[11] Brown, C., Tollefson, N., Dunn, W., Cromwell, R., & Filion, D. (2001). The Adult Sensory Profile: Measuring patterns of sensory processing. The American Journal of Occupational Therapy, 55(1), 75–82. 10.5014/ajot.55.1.75 11216370

[12] Burke, D. L., Ensor, J., & Riley, R. D. (2017). Meta-analysis using individual participant data: One-stage and two-stage approaches, and why they may differ. Statistics in Medicine, 36(5), 855–875. 10.1002/sim.7141 PMC5297998 27747915

[13] Bush, K. (1998). The AUDIT alcohol consumption questions (AUDIT-C) an effective brief screening test for problem drinking. Archives of Internal Medicine, 158(16), 1789. 10.1001/archinte.158.16.1789 9738608

[14] Chu, X., Ilyas, I. F., Krishnan, S., & Wang, J. (2016). Data cleaning: Overview and emerging challenges. Proceedings of the 2016 International Conference on Management of Data, 2201–2206. 10.1145/2882903.2912574

[15] Dimitrov, D. M. (2010). Testing for factorial invariance in the context of construct validation. Measurement and Evaluation in Counseling and Development, 43(2), 121–149. 10.1177/0748175610373459

[16] Douglas, B. D., Ewell, P. J., & Brauer, M. (2023). Data quality in online human-subjects research: Comparisons between MTurk, Prolific, CloudResearch, Qualtrics, and SONA. PLOS ONE, 18(3), e0279720. 10.1371/journal.pone.0279720 PMC10013894 36917576

[17] Dunn, W. (1997). The impact of sensory processing abilities on the daily lives of young children and their families: A conceptual model: Infants & Young Children, 9(4), 23–35. 10.1097/00001163-199704000-00005

[18] Dunn, W. (2001). The sensations of everyday life: Empirical, theoretical, and pragmatic considerations. The American Journal of Occupational Therapy, 55(6), 608–620. 10.5014/ajot.55.6.608 12959225

[19] Eysenck, H. J. (1967). Personality and extra-sensory perception. Journal of the Society for Psychical Research, 44(732), 55–71.

[20] Falkenstein, T., Sartori, L., Malanchini, M., Hadfield, K., & Pluess, M. (2025). The relationship between environmental sensitivity and common mental-health problems in adolescents and adults: A systematic review and meta-analysis. Clinical Psychological Science, 14(2), 135–157. 10.1177/21677026251348428

[21] Gray, J. A. (1982). The neuropsychology of anxiety: An enquiry into the functions of the septo-hippocampal system. Clarendon Press/Oxford University Press.

[22] Greven, C. U., Lionetti, F., Booth, C., Aron, E. N., Fox, E., Schendan, H. E., Pluess, M., Bruining, H., Acevedo, B., Bijttebier, P., & Homberg, J. (2019). Sensory processing sensitivity in the context of environmental sensitivity: A critical review and development of research agenda. Neuroscience & Biobehavioral Reviews, 98, 287–305. 10.1016/j.neubiorev.2019.01.009 30639671

[23] Gummer, T., Roßmann, J., & Silber, H. (2021). Using instructed response items as attention checks in web surveys: Properties and implementation. Sociological Methods & Research, 50(1), 238–264. 10.1177/0049124118769083

[24] Hahn, E. A., Bode, R. K., Garcia, S. F., Castel, L. D., Eisen, S. V., Bosworth, H. B., Heinemann, A. W., Rothrock, N., Cella, D., & on behalf of the PROMIS Cooperative Group. (2010). Measuring social health in the patient-reported outcomes measurement information system (PROMIS): Item bank development and testing. Quality of Life Research, 19(7), 1035–1044. 10.1007/s11136-010-9654-0 PMC3138729 20419503

[25] Hall, J. (2011). Engaging teachers and students with real data: Benefits and challenges. In C. Batanero, G. Burrill, & C. Reading (Eds.), Teaching Statistics in School Mathematics-Challenges for Teaching and Teacher Education (Vol. 14, pp. 335–346). Springer Netherlands. 10.1007/978-94-007-1131-0_32

[26] Harris, P. A., Taylor, R., Minor, B. L., Elliott, V., Fernandez, M., O’Neal, L., McLeod, L., Delacqua, G., Delacqua, F., Kirby, J., Duda, S. N., & REDCap Consortium. (2019). The redcap consortium: Building an international community of software platform partners. Journal of Biomedical Informatics, 95, 103208. 10.1016/j.jbi.2019.103208 PMC7254481 31078660

[27] Harris, P. A., Taylor, R., Thielke, R., Payne, J., Gonzalez, N., & Conde, J. G. (2009). Research electronic data capture (redcap)—A metadata-driven methodology and workflow process for providing translational research informatics support. Journal of Biomedical Informatics, 42(2), 377–381. 10.1016/j.jbi.2008.08.010 PMC2700030 18929686

[28] Hussong, A. M., Curran, P. J., & Bauer, D. J. (2013). Integrative data analysis in clinical psychology research. Annual Review of Clinical Psychology, 9(1), 61–89. 10.1146/annurev-clinpsy-050212-185522 PMC3924786 23394226

[29] Idris, K. (2018). Teaching and learning statistics in college: How learning materials should be designed. Journal of Physics: Conference Series, 1088, 012032. 10.1088/1742-6596/1088/1/012032

[30] Kessler, R. C., Andrews, G., Colpe, L. J., Hiripi, E., Mroczek, D. K., Normand, S.-L. T., Walters, E. E., & Zaslavsky, A. M. (2002). Short screening scales to monitor population prevalences and trends in non-specific psychological distress. Psychological Medicine, 32(6), 959–976. 10.1017/S0033291702006074 12214795

[31] Lamers, S. M. A., Westerhof, G. J., Bohlmeijer, E. T., Ten Klooster, P. M., & Keyes, C. L. M. (2011). Evaluating the psychometric properties of the mental health Continuum-Short Form (MHC-SF). Journal of Clinical Psychology, 67(1), 99–110. 10.1002/jclp.20741 20973032

[32] Lionetti, F., Pastore, M., Moscardino, U., Nocentini, A., Pluess, K., & Pluess, M. (2019). Sensory processing sensitivity and its association with personality traits and affect: A meta-analysis. Journal of Research in Personality, 81, 138–152. 10.1016/j.jrp.2019.05.013

[33] McCrae, R. R., & John, O. P. (1992). An Introduction to the Five-Factor Model and Its Applications. Journal of Personality, 60(2), 175–215. 10.1111/j.1467-6494.1992.tb00970.x 1635039

[34] Monroe, S. M., & Simons, A. D. (1991). Diathesis-stress theories in the context of life stress research: Implications for the depressive disorders. Psychological Bulletin, 110(3), 406–425. 10.1037/0033-2909.110.3.406 1758917

[35] Ng, W. Z., Erdembileg, S., Liu, J. C. J., Tucker, J. D., & Tan, R. K. J. (2025). Increasing rigor in online health surveys through the reduction of fraudulent data. Journal of Medical Internet Research, 27, e68092. 10.2196/68092 PMC12370263 40840500

[36] Nhem, M. K., Personette, C. M., Childers-Rockey, Z. A., Bissa, M. B. & Rioux, C. (2026). Environmental sensitivity to social environments in the prediction of mental health in adulthood: A scoping review of studies examining diathesis-stress, vantage sensitivity and differential susceptibility. Current Psychology, 45. Article 394. 10.1007/s12144-025-08919-0

[37] Peer, E., Rothschild, D., Gordon, A., Evernden, Z., & Damer, E. (2021). Data quality of platforms and panels for online behavioral research. Behavior Research Methods, 54(4), 1643–1662. 10.3758/s13428-021-01694-3 PMC8480459 34590289

[38] Pluess, M. (2015). Individual differences in environmental sensitivity. Child Development Perspectives, 9(3), 138–143. 10.1111/cdep.12120

[39] Pluess, M., & Belsky, J. (2013). Vantage sensitivity: Individual differences in response to positive experiences. Psychological Bulletin, 139(4), 901–916. 10.1037/a0030196 23025924

[40] Pluess, M., Lionetti, F., Aron, E. N., & Aron, A. (2023). People differ in their sensitivity to the environment: An integrated theory, measurement and empirical evidence. Journal of Research in Personality, 104, 104377. 10.1016/j.jrp.2023.104377

[41] Rothbart, M. K., & Derryberry, D. (1981). Development of individual differences in temperament. In M. E. Lamb, & A. L. Brown (Eds.), Advances in developmental psychology (Vol. 1, pp. 37–86). Hillsdale, NJ: Erlbaum

[42] Rioux, C., Waldron, M. E., Fulp, D., Personette, C., & Nhem, M. K. (2025). Sensory profile of the highly sensitive personality. PsyArXiv. 10.1016/j.paid.2026.113710

[43] Smith, G. (1998). Learning statistics by doing statistics. Journal of Statistics Education, 6(3), 5. 10.1080/10691898.1998.11910623

[44] Sutton, A. J., Kendrick, D., & Coupland, C. A. C. (2008). Meta-analysis of individual- and aggregate-level data. Statistics in Medicine, 27(5), 651–669. 10.1002/sim.2916 17514698

[45] Tabachnick, B. G., & Fidell, L. S. (2019). Using multivariate statistics (7th ed.). Pearson.

[46] Victorian Government Department of Human Services. (2022). Victorian Population Health Survey 2001: Selected Findings. Rural and Regional Health and Aged Care Services Division. https://www.health.vic.gov.au/sites/default/files/migrated/files/collections/research-and-reports/v/vic-population-health-survey-2001.pdf

[47] Wiberg, M. (2009). Teaching statistics in integration with psychology. Journal of Statistics Education, 17(1), 11. 10.1080/10691898.2009.11889509

[48] Williams, D. R., Yan Yu, Jackson, J. S., & Anderson, N. B. (1997). Racial differences in physical and mental health: Socio-economic status, stress and discrimination. Journal of Health Psychology, 2(3), 335–351. 10.1177/135910539700200305 22013026

