# Supplementary material for: Data from the Multi-Sensitivity Study: Sensory and Rejection Sensitivity, Mental Health, Substance Use, Discrimination, and Social Support
Source: J Open Psychol Data. 2026 Nov 2;14:8. doi: 10.5334/jopd.155 (PMC13741934; doi:10.5334/jopd.155)
Supplement: Supplementary file 1 [file jopd-14-155-pr1.pdf]

PEER REVIEW FOR "Data from the Multi-Sensitivity Study: Sensory and rejection sensitivity, mental health, substance use, discrimination, and social support"

Dear Charlie Rioux, Megan E. Waldron, Delaney C. Fulp,

After review, we have reached a decision regarding your submission to Journal of Open Psychology Data, " Data from the Multi-Sensitivity Study: Sensory and rejection sensitivity, mental health, substance use, discrimination, and social support ". Our decision is to request revisions of the manuscript prior to acceptance for publication.

Both reviewers have recommended revisions, and provided some well-specified and constructive suggestions to improve the clarity of the work presented. The full review information is included at the bottom of this email. Please note that there may also be a copy of the manuscript file with reviewer comments available once you have accessed the submission account. In summary, I don't have much to add to their comments beyond endorsing them as sensible suggestions and I encourage you to work through these systematically making any changes you see fit that would help with the clarity and impact of the work. I really look forward to reading the revised version of your work, which I imagine will not require a further round of review.

Kind regards,

Prof Thomas Rhys Evans

Reviewer 1:
Recommendation: Revisions Required

Comments to the author(s)

Dear authors, I have uploaded a Word document containing the reviewer comments (Reviewer Comments.docx) and the original manuscript with tracked changes (Sensory_DataPaper_20250729_final_reviewed.docx). Please refer to the reviewer comments document for further details.

Reviewer 2:
Recommendation: Revisions Required

Comments to the author(s)

I have reviewed the materials based on the required review guidelines for the journal, which does not include validity, importance, or writing style. In general, the content appears to be correctly described, the methods are reported in sufficient detail, and the reuse section has specific suggestions for reuse. The deposited data is in a suitable repository (OSF) but see point #3 below; given recent developments, it may make sense to change repositories. The data uses a CC-BY lisence, and (though some of the files are proprietary SPSS files) there are non-proprietary alternatives (e.g., .csv). The study does appear to follow ethical protocols and a consent form is provided. I have a few recommendations below which I have enumerated.

1. In the “Study Materials” file in your repository where the questionnaires are listed, it would be helpful to have a table of contents listing each questionnaire and the page number (and possibly citations to the sources). It was somewhat difficult to navigate this file to find which precise items corresponded to which questionnaire.

2. Relatedly, the data dictionaries are somewhat difficult to follow. Though there are data dictionaries generated in an automated fashion from SPSS (in the raw and processed folders) and from REDcap (in the study materials folder), neither of these is particularly convenient for the reader perusing the dataset. You might consider creating a single human-generated data dictionary that clearly provides:
a) Actual item content
b) Which questionnaire / construct it belongs to (when applicable)
c) How total scores are calculated, when total scores are created
d) Variable names
e) Response options
f) And overall formatting that is easy for humans to read and search

At present, the reader has to bounce between the SPSS data dictionary, the REDcap data dictionary, and the body of the text in the written article to find all of this information. Information is all present in various places, but its layout is not convenient for the end user.

3. This is optional, but as you probably know OSF is going to be retired as a repository and will not be open to new projects or edits soon enough (i.e., due to lack of funding, it will soon become read-only). You may want to consider an alternative repository so that you will be able to make edits moving forward if any errors are discovered by end users down the road.

4. On page 4 of the manuscript, you say “The survey was completed by 617 participants”. However, in the .csv file uploaded to the “Raw data” folder in your repository, there are 623 rows. Please resolve this discrepancy. That said, the final sample size of 565 reported in text does match the processed dataset.

5. The authors describe windsorizing outliers based on z-scores larger than +-3.29 on page 12. This procedure is controversial and often not psychometrically valid. Validity of the data is not a criterion for the journal, but it is probably true that most end users would not use this procedure by default; so, in that sense, it is likely unwanted data cleaning for a final processed dataset. It would be better to just provide the syntax for readers who want to employ this procedure, with the finalized dataset not incorporating it by default. This is because windsorizing is easier to employ than to reverse.
